# Linguistic markers of autism in girls: evidence of a “blended phenotype” during storytelling

**DOI:** 10.1186/s13229-019-0268-2

**Published:** 2019-03-27

**Authors:** Jaclin Boorse, Meredith Cola, Samantha Plate, Lisa Yankowitz, Juhi Pandey, Robert T. Schultz, Julia Parish-Morris

**Affiliations:** 1 Lehigh University, College of Education, 111 Research Drive, Bethlehem, PA 18015 USA; 2 Center for Autism Research, Children’s Hospital of Philadelphia, 2716 South St, Philadelphia, PA 19104 USA; 3 Department of Psychology, University of Pennsylvania, Philadelphia, USA; 4 Department of Psychiatry, Perelman School of Medicine, University of Pennsylvania, Philadelphia, USA; 5 Department of Pediatrics, Perelman School of Medicine, University of Pennsylvania, Philadelphia, USA

**Keywords:** Autism spectrum disorder, Storytelling, Narratives, Natural language processing, Social cognition, Word choice, Mentalizing, Sex differences

## Abstract

**Background:**

Narrative abilities are linked to social impairment in autism spectrum disorder (ASD), such that reductions in words about cognitive processes (e.g., *think*, *know*) are thought to reflect underlying deficits in social cognition, including Theory of Mind. However, research suggests that typically developing (TD) boys and girls tell narratives in sex-specific ways, including differential reliance on cognitive process words. Given that most studies of narration in ASD have been conducted in predominantly male samples, it is possible that prior results showing reduced cognitive processing language in ASD may not generalize to autistic girls. To answer this question, we measured the relative frequency of two kinds of words in stories told by autistic girls and boys: nouns (words that indicate object-oriented storytelling) and cognitive process words (words like *think* and *know* that indicate mentalizing or attention to other peoples’ internal states).

**Methods:**

One hundred two verbally fluent school-aged children [girls with ASD (*N* = 21) and TD (*N* = 19), and boys with ASD (*N* = 41) and TD (*N* = 21)] were matched on age, IQ, and maternal education. Children told a story from a sequence of pictures, and word frequencies (nouns, cognitive process words) were compared.

**Results:**

Autistic children of both sexes consistently produced a greater number of nouns than TD controls, indicating object-focused storytelling. There were no sex differences in cognitive process word use in the TD group, but autistic girls produced significantly more cognitive process words than autistic boys, despite comparable autism symptom severity. Thus, autistic girls showed a unique narrative profile that overlapped with autistic boys *and* typical girls/boys. Noun use correlated significantly with parent reports of social symptom severity in all groups, but cognitive process word use correlated with social ability in boys only.

**Conclusion:**

This study extends prior research on autistic children’s storytelling by measuring sex differences in the narratives of a relatively large, well-matched sample of children with and without ASD. Importantly, prior research showing that autistic children use fewer cognitive process words is true for boys only, while object-focused language is a sex-neutral linguistic marker of ASD. These findings suggest that sex-sensitive screening and diagnostic methods—preferably using objective metrics like natural language processing—may be helpful for identifying autistic girls, and could guide the development of future personalized treatment strategies.

In this paper, our terminology is drawn from World Health Organization definitions, such that the word “sex” refers to genetic makeup, and “gender” refers to a socio-cultural construct [111]; we use the words “girl” and “boy” to refer to biological sex. We recognize that narratives may be spoken, signed, or written; in this study, we explore spoken narratives. In line with preferences expressed by self-advocates within the autistic community (L. [15, 37]), this paper uses identity-first language and refers to participants diagnosed with autism as autistic girls and boys.

## Introduction

Recent efforts to quantify clinical heterogeneity in autism spectrum disorder (ASD) indicate that verbal autistic girls and women behave differently than male counterparts, even when matched on social symptom severity [38]. Certain behaviors, like mimicking other people’s facial expressions or gestures, making eye contact, and memorizing social scripts may serve as “camouflage” for social impairments [63], and are thought to be utilized more often by autistic girls and women than autistic boys and men [49]. Sex-specific differences in autistic behaviors, including camouflaging, are not explicitly measured by current gold standard diagnostic instruments [87], leading to concerns that girls are systematically under-diagnosed compared to boys [68]. Sex is a core biological difference that impacts children’s experiences before, during, and after ASD symptoms emerge [25], so understanding the effects of sex on ASD expression has important implications for diagnostic and clinical practice. For instance, quantifying the precise nature of sex differences in ASD could help clinicians develop personalized interventions that are more effective than a one-size-fits-all approach to autism treatment.

Direct behavioral measurement is the primary method for diagnosing ASD [69], but recent evidence points to a variety of “autistic behaviors” that present differently in girls. For example, atypical or reduced gesturing is common in ASD [32, 52], but empirical studies suggest that verbal autistic girls produce gestures that are more vibrant and noticeable than autistic boys [95]. Researchers have argued that autism is associated with unusual verbal disfluency patterns [47, 51, 65], but autistic girls produce disfluency patterns that are sex-typical, and measurably distinct from the disfluency patterns of autistic boys [82]. Autism is associated with diminished social attention [60], but recent evidence from infrared eye tracking suggests that autistic girls may look more at faces than autistic boys (Harrop et al., under review). On the playground, autistic girls are more likely to hover near groups of other girls, whereas autistic boys are more likely to be isolated [35]. As adults, autistic women show greater discrepancies between outward symptoms of ASD and their own internal experiences [64]. Taken together, these differences suggest that the behavioral symptoms of ASD manifest differently in girls and women than they do in boys and men.

For verbal individuals, language is an important pathway to friendships, romantic relationships, jobs, and overall quality of life. Given population sex differences in a variety of linguistic domains [75, 76, 108], and the core dimensions of social communication that are used to diagnose ASD [1], understanding similarities and differences in language produced by autistic boys and girls could shed light on sex-specific differences in the clinical presentation of autism. In this study, we focus on sex and diagnostic group differences in the language children use during a brief storytelling task.

### Narratives

Storytelling is an ancient social art that hinges on interpersonal skills. Reliance on oral histories has diminished over time, but brief daily storytelling is preserved as a central component of communal living. Even the simplest question, “How was your day?” provides an opportunity for short narratives to strengthen interpersonal connections. Storytelling is ubiquitous, and the basic elements of storytelling are acquired by most children in early childhood [39, 85]. However, storytelling relies on much more than vocabulary and grammar. In fact, successful storytellers leverage a rich array of skills, including working memory [18], executive function [20], and a sense of social appropriateness, or knowing how much information to provide to different kinds of listeners [105]. Practical language skills that use social context to facilitate effective communication (i.e., pragmatic language abilities) are centrally important for storytelling. For example, speakers must monitor whether listeners are engaged, and whether they understand the story. They must watch facial expressions and interpret nonverbal cues to guide them to explain further, pause, or otherwise act to get the listener back on track. Given this important pragmatic dimension to storytelling, it is unsurprising that narrative competence is closely related to social ability [16, 104].

### Narrative skills in autism

Pragmatic language skills are universally impaired in ASD [1], with a large body of research showing that the narratives of autistic adults [5, 8, 10, 66, 74] and children differ from typically developing (TD) peers in a variety of measurable ways [3]. These differences include impoverished event explanations [21, 58, 101], reduced story structure complexity [83], reduced coherence [71], reduced syntactic complexity, more ambiguous pronouns, fewer story grammar elements [6], poorer inferencing, and a tendency to include extraneous information [72]. In adolescence, even “optimal outcome” individuals who no longer meet ASD criteria show subtle language differences during narrative tasks, with higher rates of self-correction and idiosyncratic speech compared to controls [19, 33, 56, 100]. Importantly, careful matching on language ability does not ameliorate diagnostic group differences; a number of studies found that the narratives of autistic children still differ on structural, evaluative, and global narrative features, including shorter stories and reduced causal statements, suggesting that other factors besides language ability must explain performance discrepancies [36, 57, 58, 98, 101].

Narratives produced by children with ASD not only differ from narratives produced by typically developing peers, but from other clinical groups as well. Compared to narratives produced by children with specific language impairment (SLI), autistic children’s narratives show weaknesses in areas that rely on perspective-taking, such as mental state language (e.g., *think*, *know*, *believe*), referencing, and relevancy [28]. However, children with ASD and comparison children with SLI produce similarly simplistic and semantically lean narratives that omit important story elements, relative to TD controls [79] and both groups make more ambiguous references during storytelling [78]. This suggests that social impairment and language deficits result in distinct narrative profiles [41]. Compared to children with attention deficit/hyperactivity disorder (ADHD), autistic children refer less to cognitive states and provide less coherent narratives. However, both groups leave out key story components and produce shorter narratives than TD peers [91]. Because individuals with ASD narrate in a way that is unique to their diagnostic group, narrative generation and retelling tasks are viewed as clinical tools that shed light on various aspects of atypical development [13, 28, 62, 73].

As in TD children, research shows that narrative ability is far from a standalone skill in autistic children; rather, it has been linked to a broader set of social, cognitive, and communicative abilities, including Theory of Mind [4, 98], working memory [62], emotional understanding [70], and conversational competence [4, 98]. The relative centrality of narrative ability for social competence in ASD [104], as well as for academic success [99], has made it a popular intervention target [44, 84, 110].

### Word choice during narration

Words are necessary for conveying the contents of a story. In addition, word choice and frequency shed light on what a speaker finds important enough to describe [103], and thus may be interpreted as a measure of preference or motivation [59]. Word choice is particularly interesting in ASD, as autistic individuals regularly produce idiosyncratic words or phrases during narratives [27, 70], when describing videos [59], and during clinical interviews [80, 81]. Among the most widely studied word-based differences in ASD are (1) concrete/literal language, generally reported to be more common in ASD than matched controls, and (2) cognitive/mental state words, often described as diminished and reflecting poor Theory of Mind in ASD.

### Concrete/literal language

The first published accounts of verbal autistic children included descriptions of overly formal and pedantic language [2, 53, 54], which made children sound like “little professors.” Thinking and speaking patterns described as “concrete” and “literal” soon followed [48, 88], as well as reports that autistic individuals have difficulty understanding irony, sarcasm, metaphor, and deceit [7, 54, 92]. Research shows that concrete words are more likely to be nouns than any other word class [11], although some nouns are abstract (e.g., “justice”). In the present study, we use the number of nouns produced by children during narratives to indicate object orientation or concreteness, and aim to replicate prior research showing that children with ASD produce language that is more concrete and object-focused than matched typical peers.

### Cognitive process words

Autistic children’s narratives have been found to contain fewer cognitive process words like *think* and *know* (also referred to as mentalizing words or internal state language), than narratives produced by typical peers [9, 14, 21, 55, 83]. Reduced reliance on cognitive process words is argued to index diminished social cognition in autism [9, 59], and indeed, the proportion of cognitive process words produced during autistic children’s narratives predicts their ability to understand the thoughts and feelings of others (Theory of Mind) [98]. However, unexplained heterogeneity in autistic children’s cognitive process language still exists across samples and tasks, as some studies do not report this effect [58, 96, 102]. Possible explanations for these mixed findings include heterogeneous sampling and failure to consider the influence of relevant factors like biological sex on word choice during storytelling. In this study, we test the hypothesis that children with ASD produce fewer words about cognitive processes than matched peers, with an eye toward potential moderating effects of biological sex on word choice.

### Sex differences in narration

From an early age, typically developing girls and boys have different narrative experiences. Parental narratives directed at girls include more references to emotions and internal states than narratives directed toward boys [43]. Subsequently, girls tell narratives that are distinct from boys’ narratives [12, 17, 67], including longer narratives that are more emotionally laden and more likely to reference others’ internal states [77, 97]. However, it is largely unknown whether the narratives of boys and girls with ASD also differ from one another.

To our knowledge, one study has examined sex differences in the narratives of children with ASD. In this small study, German-speaking autistic girls (*N* = 11) used more internal state language than autistic boys when telling a story from a wordless picture book [55]. However, the clinical autism symptom severity of the boys and girls was not reported, leaving open the possibility that autistic girls were less severely socially impaired, or more socially motivated, than autistic boys. Many otherwise large narrative studies included insufficient numbers of girls with ASD to assess sex differences in this domain [42, 66]. Due to this paucity of research, current narrative interventions are not sex-sensitive. A lack of sex-sensitive narrative interventions is especially problematic in light of recent research suggesting that storytelling is a critical social medium for school-aged autistic girls who experience peer rejection when they violate storytelling norms [34].

### The current study

This study explores sex differences in the narrations of autistic girls and boys matched on age, intelligence quotient (IQ), and social challenges. In particular, we focus on the relative frequency of two kinds of words: nouns (words that indicate object-oriented storytelling and tend to be concrete) and cognitive process words (words like *think* and *know* that indicate cognitive orientation). First, based on prior research suggesting that the speech of autistic children is often literal and dominated by concrete words [2, 48, 53, 88] and nouns are likely to be concrete [11], we expect a main effect of diagnosis on noun use. Specifically, we expect that autistic children will produce more nouns (labels for objects or characters) in their narrations compared to TD children. Second, given population sex differences in the production of cognitive process words during narrations [77, 97] and emerging research suggesting that autistic girls produce more of these words than autistic boys across a variety of tasks, including narratives [45, 55], we expect a main effect of sex on cognitive process words, such that girls use more cognitive process words than boys. Finally, consistent with prior research showing an inverse relationship between autism symptoms and mentalizing words [98], we hypothesized that cognitive word use would negatively correlate with social impairment across the sample as a whole.

## Methods

### Participants

The sample included 102 children, one group with typical development (TD) and one group with a clinical diagnosis of ASD (Table 1). Participants were drawn from research studies that administered Module 3 of the Autism Diagnostic Observation Schedule-2nd Edition (ADOS-2; [69]) to native English speakers. Parents of participants provided written informed consent to participate in this study, overseen by the Institutional Review Board of the Children’s Hospital of Philadelphia, and all participants consented to the use of their audio recordings for future research. Participants were included if they had full-scale, verbal, and nonverbal IQ estimates > 79 on a standardized intelligence test, and were able to independently complete “The Fisherman and the Cat” narrative task (described below). Diagnoses were made by expert PhD-level clinicians using the clinical best estimate approach, with support from a research-reliable administration of the ADOS-2 [69]. Study visits included ADOS-2 administration, a cognitive assessment, the Social Communication Questionnaire (SCQ; [94], and the Social Responsiveness Scales [30], as part of a larger battery of research tasks. Families were compensated for their time.

**Table 1:** Demographic and clinical characteristics of participants (means and standard deviations, in addition to minimum and maximum values)

	ASD (*N* = 62)		TD (*N* = 40)		Effects		
Sex ratio	21 f, 41 m (66% male)		19 f, 21 m (53% male)		χ^2^ = 1.37, *p* = .24		
Race	Black/African American: 1		Black/African American: 13				
	White/Caucasian: 53		White/Caucasian: 20				
	Asian or Pacific Islander: 3		Asian or Pacific Islander: 2				
	Multiracial: 4		Multiracial: 5				
	Other: 1		Other: 0				
Maternal education (in years)	≤ 12: 5%		≤ 12: 0%		χ^2^ = 2.03, *p* = .36		
	13–16: 53%		13–16: 58%				
	17+: 35%		17+: 38%				
	Not reported: 6%		Not reported: 5%				
	Female	Male	Female	Male	Sex	Dx	Sex in ASD
Age (years)	10.77 (1.54)	9.96 (1.93)	10.33 (2.62)	10.26 (2.56)	*p* = .26	*p* = .98	*p* = .10
	9.0–14.0	7.0–14.0	6.6–15.2	5.8–15.1	*d* = .23	*d* = .004	*d* = .44
Full-scale IQ	108.58 (9.63)	105.95 (11.94)	105.68 (14.67)	110.10 (10.50)	*p* = .90	*p* = .62	*p* = .39
	92–130	80–131	86–134	86–127	*d* = .03	*d* = .10	*d* = .23
Verbal IQ	108.95 (11.35)	105.46 (11.71)	108.42 (16.34)	107.52 (10.33)	*p* = .34	*p* = .70	*p* = .27
	87–134	83–127	80–148	86–130	*d* = .20	*d* = .08	*d* = .30
Non-verbal IQ	108.14 (11.93)	106.54 (12.89)	102.63 (14.45)	109.33 (12.88)	*p* = .49	*p* = .80	*p* = .64
	85–130	83–140	81–132	89–145	*d* = .14	*d* = .05	*d* = .13
ADOS-2 CSS total	6.38 (2.64)	6.71 (2.37)	1.16 (0.50)	1.43 (0.68)	*p* = .45	*p* < .001	*p* = .62
	1–10	3–10	1–3	1–3	*d* = .09	*d* = 2.70	*d* = .13
ADOS-2 SA	6.24 (2.51)	6.71 (2.39)	1.68 (0.95)	2.14 (0.91)	*p* = .25	*p* < .001	*p* = .48
	3–10	3–10	1–4	1–3	*d* = .16	*d* = 2.30	*d* = .19
ADOS-2 RRB	6.95 (2.60)	6.93 (2.50)	1.42 (1.26)	1.67 (1.71)	*p* = .85	*p* < .001	*p* = .97
	1–10	1–10	1–5	1–7	*d* = .03	*d* = 2.47	*d* = .01
SRS T-score	80.39 (14.90)	75.76 (15.63)	45.84 (6.64)	45.61 (8.05)	*p* = .33	*p* < .001	*p* = .30
	58–114	45–111	35–55	35–65	*d* = .13	*d* = 2.50	*d* = .30
SCQ total	20.29 (5.21)	20.27 (7.01)	2.21 (2.95)	2.50 (1.73)	*p* = .92	*p* < .001	*p* = .99
	9–31	5–33	0–10	0–6	*d* = .01	*d* = 3.41	*d* = .003

Three autistic girls and 1 autistic boy had missing SRS T-scores; SCQ scores were missing for 1 TD boy. *CSS* ADOS-2 calibrated severity score, *SA* social affect, *RRB* repetitive behaviors/restricted interests. Chi-squared tests with Yates’ continuity correction tested for diagnostic group differences in sex ratio and maternal educational attainment. *P* values and Cohen’s *d* values for main effects of sex and diagnosis are shown (simple linear model with the whole sample; there were no significant interactions), as well as *p* and Cohen’s *d* values of sex differences in the ASD group only

### Measures

All children were administered the Autism Diagnostic Observation Schedule-Second Edition (ADOS-2; [69]) Module 3, which requires fluent verbal skills. ADOS-2 scores comprise two domains, Social Affect and Restricted and Repetitive Behaviors, which combine to create the overall score [50]. The Social Communication Questionnaire (SCQ; [94]) was filled out by parents, usually the participant’s mother, prior to the clinical visit (*N* = 1 missing). The “Lifetime” score of the SCQ was used in this study, which includes items assessing behavior when the child was 4 to 5 years old, along with symptoms ever demonstrated across the participant’s life, rather than only current behavior [22]. The Social Responsiveness Scale, 2nd Edition-Parent Report [30] was completed by parents at the time of the clinical visit (*N* = 4 missing). The Social Responsiveness Scale-2nd Edition (SRS-2) was designed for use in the general population, and includes sex-normed T-scores. The SRS-2 and SCQ were chosen as primary estimates of autism symptom severity and social impairment for correlations with language (rather than ADOS-2 severity scores) because our language sample was drawn from the ADOS-2.

All participants received a cognitive assessment at their visit. Clinicians administered either the Differential Ability Scales-2nd Edition (*N* = 57; DAS-II; [40]), the Weschler Abbreviated Scale of Intelligence-2nd Edition (*N* = 38; WASI-II; [106]), the Abbreviated Stanford-Binet Intelligence Scales-5th Edition (*N* = 5; SB5; [90]), or the Weschler Intelligence Scale for Children-5th Edition (*N* = 2; WISC-V; [107]). These assessments were standardized and reduced to a single cognitive estimate, and verbal and nonverbal subscores, by an expert licensed neuropsychologist (J. Pandey) to allow for comparison among tests.

### Narrative sample

**Fig. 1:**
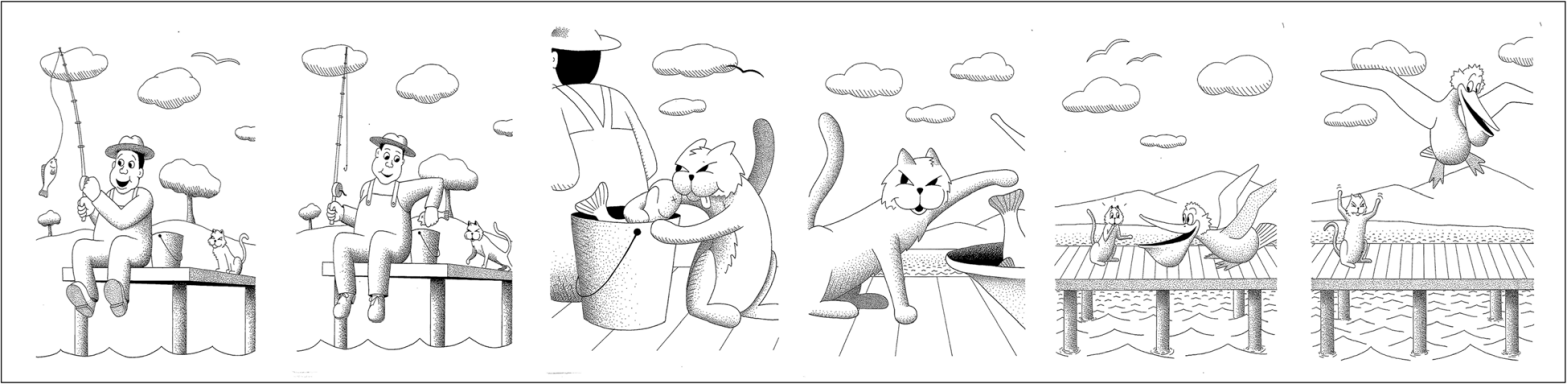
“The Fisherman and the Cat” from ADOS-2 Module 3

Narrative samples were taken from research-reliable administrations of the ADOS-2 Module 3 recorded at the Center for Autism Research at the Children’s Hospital of Philadelphia. Participants were asked to tell a story out loud while looking at a series of six pictures about a fisherman and a cat (Fig. 1). This ADOS-2 task involves a first telling (while looking at the pictures) and a second telling (with no pictures). For this study, we only examine the first telling. For some children, clinicians take an active role in the narration (e.g., clinicians sometimes demonstrate how to narrate by describing a picture themselves first). These cases, wherein clinicians took an active role in the narration, were not included the current sample of 102. Although tasks were most often administered in order, the ADOS-2 manual specifies that clinicians have the flexibility to change task administration order if they feel it is appropriate.

### Language processing

Audio recordings were separated from video recordings of the ADOS-2, and orthographically transcribed by reliable annotators who were unaware of participants’ diagnostic status and study hypotheses. Approximately 20% of the sample was transcribed twice by independent transcribers (20 recordings), with word level reliability averaging 92% (see [80, 81] for a review of transcription methods). Transcripts were processed using the Quantitative Discourse Analysis Package (qdap; [89]) for nouns and the Linguistic Inquiry and Word Count program (LIWC 2015; [103]) for cognitive process words (cogproc; Table 2).

**Table 2:** Example cognitive process words from the LIWC dictionary

Want	Need	Decide
Think	Know	Wonder
Feel	Would	Could
Believe	Guess	Depend
Realize	Reason	Suppose

### Dependent variables

Two primary variables were analyzed: (1) the number of nouns produced during the narration (concrete orientation) and (2) the number of cognitive process words produced during the narration (cognitive orientation). Preliminary analyses revealed significant differences in word count by diagnostic group and sex (significant main effects, TD > ASD, girls > boys, *p*s < .001, no significant interaction; Table 3), so both dependent variables were calculated per 100 words.

**Table 3:** Means and standard deviations of total words produced during the narration by diagnostic group (ASD, TD) and sex (Male, Female) overall and within each subgroup

Dx	M (SD)	Sex	M (SD)	Dx by sex	M (SD)
TD	75.23 (26.45)	Female	74.90 (28.58)	TD Female	82.16 (33.71)
				TD Male	68.95 (15.94)
ASD	63.44 (24.97)	Male	63.65 (23.53)	ASD Female	68.33 (21.78)
				ASD Male	60.92 (26.36)

### Statistical approach

Two generalized linear models (GLM, family = *Poisson*) assessed effects of diagnostic group (TD = 0, ASD = 1) and sex (Female = 0, Male = 1) on concrete orientation and cognitive orientation (“glm” from the “stats” package in R [86]), after controlling for maternal education, verbal IQ, and chronological age. Interactions between diagnostic group and sex were tested in each model, but were dropped for parsimony if the interactive effect was not a significant predictor. Estimates, *z* values, and *p* values are reported for primary dependent variables. Estimated marginal means (EMM; “emmeans” package in R) accounting for control variables are used for pairwise comparisons; *p* values were corrected using Tukey’s HSD. Effect sizes for GLM are reported as standardized mean differences (SMD; appropriate for *Poisson* distributions and interpreted in standard deviations [31]), and Cohen’s *d* for simple mean differences (e.g., Table 1). Following Cohen [26], SMD or *d* = 0.2 is considered a “small” effect, SMD or *d* = 0.5 a “medium” effect, and SMD or *d* = 0.8 a “large” effect [26]. Spearman correlations (*rho*) tested relationships between cognitive vs. concrete orientation and autism symptom severity.

## Results

### Concrete orientation (number of nouns)

**Fig. 2:**
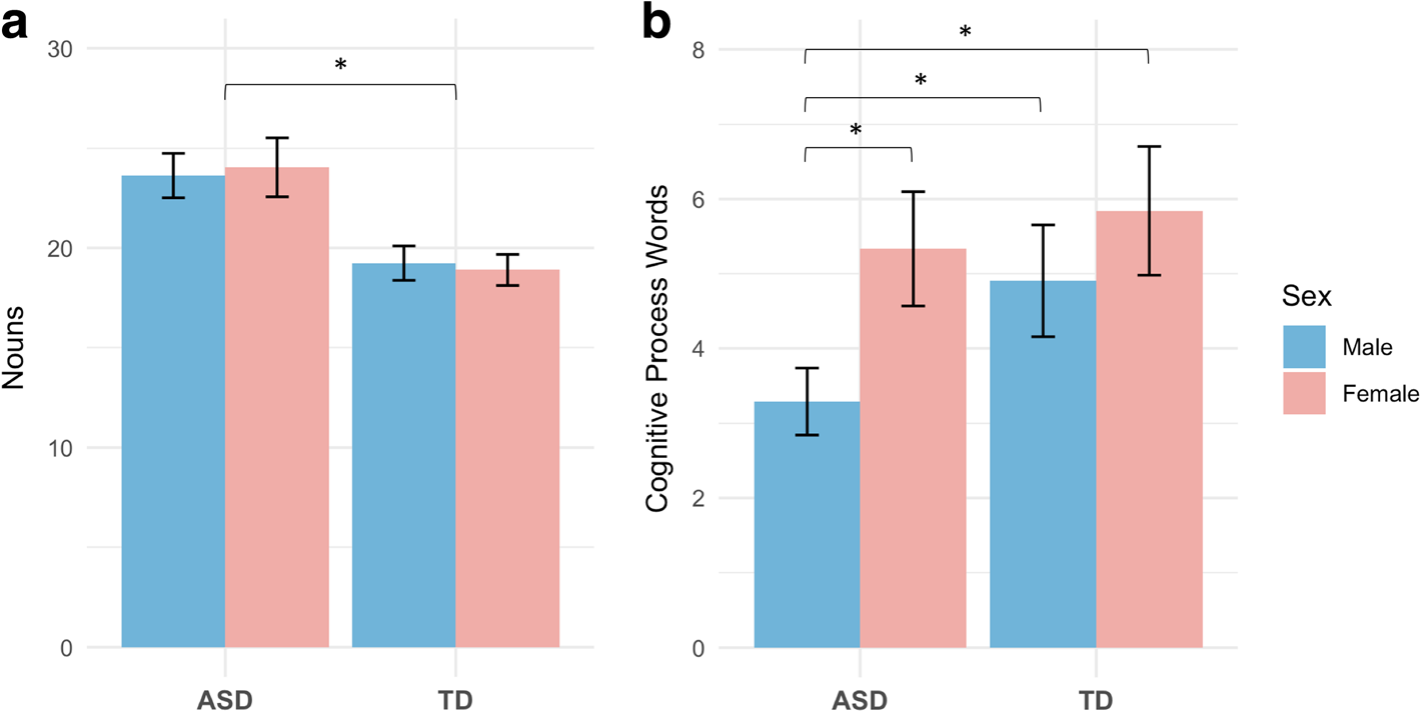
Means and standard errors of concrete orientation (**a**) and cognitive orientation (**b**) by diagnostic group (ASD, TD) and sex (Male, Female); * *p* < .05

There was no significant diagnosis by sex interaction on the number of nouns produced during the narrative task, so the interaction was dropped from the model. A model including sex and diagnosis revealed a main effect of diagnostic group: autistic children produced significantly more nouns per 100 words (EMM = 23.44) than TD participants (EMM = 18.66; *z* = 4.88, *p* < .001, SMD = 1.08; Fig. 2a). There was no significant main effect of sex on the number of nouns produced.

### Cognitive orientation (number of cognitive process words)

There was a significant interactive effect of sex and diagnostic group on cognitive process words (*z* = − 2.10, *p* = .04, SMD = .81). Removing diagnosis from the model revealed a main effect of sex, such that girls produced significantly more cognitive process words (EMM = 5.61) than boys (EMM = 4.03; *z* = 3.86, *p* = .0001, SMD = .76). There was also a main effect of diagnostic group, such that TD children produced significantly more cognitive process words (EMM = 5.51) than autistic children (EMM = 4.93; *z* = 2.27, *p* = .02, SMD = .45). Pairwise comparisons of estimated marginal means calculated from the full model (including the sex * diagnosis interaction) revealed that whereas TD girls and boys produced statistically similar numbers of cognitive process words during their narrations (TD girls: EMM = 5.79, TD boys: EMM = 4.97, *z* = − 1.07, *p* = .71, SMD = .33), autistic girls produced significantly more cognitive process words (EMM = 5.92) than autistic boys (EMM = 3.37; *z* = 4.10, *p* = .0002, SMD = 1.07). Autistic girls’ production of cognitive process words did not significantly differ from TD girls, nor from TD boys. However, autistic boys produced a significantly smaller number of cognitive process words than TD boys (*z* = 2.84, *p* = .02, SMD = .70; Fig. 2b).

### Correlations with social ability and autism symptoms

**Fig. 3:**
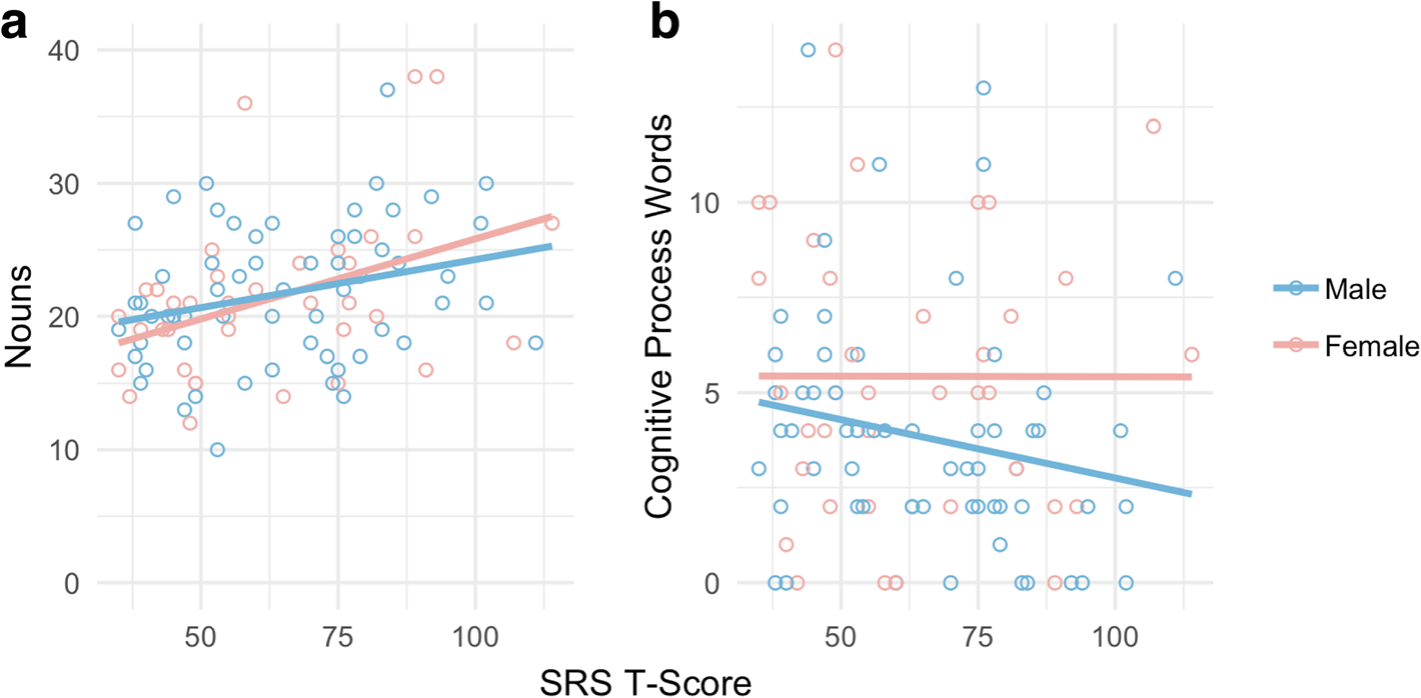
Correlations between parent ratings of social ability and (**a**) concrete orientation (number of nouns), and (**b**) cognitive orientation (number of cognitive process words) by sex, as measured by SRS-2 T-scores (higher scores indicate greater social impairment)

A cross the sample as a whole, concrete orientation (i.e., the number of nouns produced during the narration) correlated with parent ratings of social impairment (SRS-2: Spearman’s *rho* = .35, *p* = .0005) and autism symptomatology (SCQ: Spearman’s *rho* = .34, *p* = .0006). These associations remained significant when examined separately within girls (SCQ: Spearman’s *rho* = .40, *p* = .01; SRS-2: Spearman’s *rho* = .40, *p* = .01) and boys (SCQ: Spearman’s *rho* = .28, *p* = .03; SRS-2: Spearman’s *rho* = .29, *p* = .02), but did not reach significance within separate diagnostic groups. Cognitive orientation (i.e., the number of cognitive process words produced during the narration) was not significantly associated with social symptom scores in the overall sample, nor in separate diagnostic groups. However, cognitive process words were significantly negatively associated with SRS-2 scores (Spearman’s *rho* = −.27, *p* = .04) and SCQ scores (Spearman’s *rho* = −.28, *p* = .03) in boys, indicating that boys who were rated as less socially impaired by their parents also produced more cognitive process words during the narrative task. These relationships were not present in girls, suggesting a disconnect between linguistic markers of cognitive orientation and parental perceptions of social ability in this subgroup (Fig. 3).

## Discussion

Autistic children have been widely reported to use fewer words about cognitive processes and more concrete (object-focused) language than typically developing peers. The results reported here replicate the latter finding; children with ASD—regardless of sex—used significantly more nouns than TD children during a narrative task, with a large effect size. This is important, as elevated noun use by both sexes suggests that a concrete, object-oriented narrative style may be a core linguistic marker of autism. Understanding fundamental differences associated with ASD that persist in both sexes could inform the hunt for digital markers of treatment efficacy, while serving as scalable clinical characterization tools in large genetic studies where lab-based phenotyping is prohibitively expensive.

In contrast, we found that reliance on cognitive processing words differed by sex in ASD, such that cognitive process word production was significantly reduced in autistic males *only*. Diminished internal state language, including cognitive process words, has been interpreted as reflecting Theory of Mind impairments in ASD [9, 59], but prior studies rarely included sufficient numbers of girls to assess whether typical sex differences in social cognition [23, 93] and narratives [77, 97] persist in autistic boys and girls separately. Our finding, that cognitive process word deficits during storytelling are largely specific to males with ASD, suggests that historical reliance on predominantly male samples may have led to generalizations about narration in ASD that do not apply to girls (although this study still includes a smaller sample of girls than boys, and warrants replication). We anticipate that the present study will spark important future research efforts aimed at identifying points of convergence and divergence in the linguistic patterns of autistic girls and boys relative to each other and to same-sex peers.

Autistic girls in our study produced narratives that contained significantly more cognitive process words than autistic boys, but their narratives nevertheless differed from TD girls’ narratives—and were similar to autistic boys—in important ways. Unlike TD girls, autistic girls produced relatively high rates of concrete, object-oriented words during their narratives, suggesting greater-than-average attunement to objects (a pattern noted in the earliest descriptions of autistic language [2, 53]). Upon closer examination, we found that some participants—male and female—had highly specific naming preferences for objects or characters in the story (e.g., *pelican*, *seagull*, *penguin* for “bird”) and wanted to land on the “correct” label. Exploring the phenomenon of labeling specificity in ASD is a promising future research direction.

Girls and boys in our sample were equally affected by ASD symptoms (according to both clinical judgment and parent report), which is an important strength of this study compared to prior research on sex differences in autistic narration [55]. Thus, our results suggest that while social impairment and mentalizing language are linked in autistic boys [98], they may be less tightly coupled in autistic girls. This hypothesis is supported by our correlation analysis, showing that social impairment is significantly related to cognitive process words in boys only—there is no significant correlation in girls. This lack of relationship between cognitive process word use and social impairment in girls may be due to effortful linguistic compensation on behalf of autistic girls. For example, describing internal states could be a learned behavior that normalizes how autistic girls are perceived relative to typical peers, masking internal social struggles and serving as “linguistic camouflage.”

Linguistic camouflage is one explanation for the pattern of results observed in this study, but if the girls did engage in masking, they were only partially successful. Autistic girls showed a unique mixed narrative style that was similar to autistic boys in one domain (nouns) and similar to typical girls and boys in another domain (cognitive process words). This “blended phenotype” may make it especially challenging to screen, refer, and make community diagnoses of verbally fluent autistic girls. Importantly, the girls in our sample had symptoms that were obvious enough to warrant referrals for autism evaluations and were ultimately diagnosed with ASD. It is therefore possible (and perhaps even likely) that this sample does not represent the full spectrum of girls who engage in more successful camouflaging throughout the teen years and into adulthood. Importantly, the current study does not directly measure *why* autistic girls show fewer cognitive process word deficits compared to autistic boys; in addition to the camouflage hypothesis, girls could have innate or acquired differences in cognitive orientation or social motivation that could lead to greater attention to cognitive processes [24] despite comparable impairment in social functioning. Future studies should prospectively follow girls’ and boys’ social and language development to elucidate when and how these differences emerge.

### Limitations

This study has significant strengths, including a relatively large sample of verbal girls with ASD and a well-matched TD control group, but it also has some limitations. First, while our task (telling a story from pictures) is a common narrative probe that relates to social cognition (e.g., Theory of Mind; [98]), it is still semi-structured. It is unknown whether the current findings will generalize to everyday conversations. Second, although participant groups were matched on full-scale IQ, verbal IQ, and non-verbal IQ, and all received Module 3 of the ADOS-2 (designed for school-aged children with verbal fluency), we did not administer a targeted language assessment like the CELF-5 [109]. This limitation makes it difficult to pinpoint whether subtle differences in the language profiles of boys and girls may have impacted their word choice, and is a promising area for future research. Third, we did not include participants who required significant help understanding or completing the narrative task, so the results reported here are expected to apply to individuals with verbal comprehension and production abilities in the average range. Fourth, executive function (EF) has been shown to impact spoken narratives [20] and we did not include an explicit EF measure in our battery. However, the influence of EF and working memory is likely diminished in this task because participants were looking at a picture while telling the story. Fifth, this study did not ask whether autistic children produce fewer abstract words overall (across semantic categories), or whether our result is specific to cognitive process words. This is a promising area for future research. Finally, to count the number of cognitive process words produced by participants in this task, we used a list provided by Linguistic Inquiry and Word Count software (LIWC 2015; [103]). Other studies may have counted different words as part of a broader, narrower, or slightly modified definition of mentalizing words or internal state language, leading to contrasting findings. Despite this limitation, one benefit of using a standardized list is that subsequent research can use the same tool to assess whether the effects reported here are reproducible and generalizable (e.g., to a different age group).

### Implications and future directions

Prior research on word choice during autistic children’s narratives either sampled primarily boys, compared ASD groups that were disproportionately male to typically developing groups with a more balanced sex ratio, or did not report sex at all. However, the assumption has been that for ASD, differences found in primarily male samples are generalizable to all autistic children, despite well-documented sex differences and gendered early experiences that affect children prior to an official diagnosis of ASD [25, 46]. This study therefore has implications for re-evaluating prior research, suggesting that “established” findings might be fruitfully re-explored with an eye toward possible hidden sex differences.

The most appropriate benchmark for measuring a person’s social behavior is another person matched on a variety of basic features including age and sex [61]. We thus join other researchers in calling for a wider range of traditional screening and diagnostic tools to consider sex-sensitive revisions or the inclusion of sex-based norms (see SRS-2 for an example of sex-based norms; [29]). Furthermore, our research suggests that objective technology-based measurement tools (e.g., natural language processing) could prove useful for extracting information from clinical interviews, including subtle differences that are difficult to identify using the human ear alone. For example, elevated noun use by autistic girls may not be obvious to a listener when those same girls also produce high rates of cognitive words, but an algorithm could detect this pattern. Thus, sex-normed diagnostics and technology-based decision support tools could help identify autistic girls earlier and better track the emergence of ASD in all children.

The present study contributes new evidence to recent efforts aimed at understanding why girls with ASD may meet clinical criteria for diagnosis, but still fail to be diagnosed with ASD and receive intervention [38, 68, 87]. Characterizing the subtle linguistic patterns that differentiate girls with and without ASD could sharpen our conceptualization of ASD in girls, and potentially support detection. In addition, understanding how storytelling differs in children with ASD—and whether the differences are universal or sex-specific—could help identify new intervention targets that are personalized to each child’s profile of strengths and weaknesses. The effects described in this study are medium-to-large, but likely represent a tiny subset of the many subtle differences that have a profound impact on how autism manifests across diverse individuals and contexts.

## Conclusion

This study extends prior research on autistic children’s storytelling by elucidating sex differences in the narratives of a relatively large, well-matched sample of children with and without autism spectrum disorder. Our results suggest that object-focused storytelling is a sex-neutral linguistic marker of ASD, and prior research showing that autistic children use fewer cognitive process words is true for boys only. Specifically, our finding that autistic girls’ narratives differ from autistic boys’ narratives in the domain of cognitive process words—but not in the area of nouns—adds to growing evidence that while girls with ASD differ from boys with ASD in measurable ways, they also retain core differences that could represent the “essence” of autism. We propose that future sex-sensitive screening, characterization, and diagnostic methods, preferably using objective metrics like natural language processing, could be helpful for identifying autistic girls and devising personalized treatment strategies.

## Abbreviations

ADOS-2: Autism Diagnostic Observation Schedule-2nd Edition
ASD: Autism spectrum disorder
EF: Executive function
EMM: Estimated marginal mean
IQ: Intelligence quotient
LIWC: Linguistic Inquiry and Word Count
M: Mean
SCQ: Social Communication Questionnaire
SD: Standard deviation
SRS-2: Social Responsiveness Scale-2nd Edition
TD: Typically developing
